# Decreased renal function among children born to women with obstructed labour in Eastern Uganda: a cohort study

**DOI:** 10.1186/s12882-024-03552-8

**Published:** 2024-03-28

**Authors:** David Mukunya, Faith Oguttu, Brendah Nambozo, Ritah Nantale, Brian Tonny Makoko, Agnes Napyo, Josephine Tumuhamye, Solomon Wani, Prossy Auma, Ketty Atim, Doreck Nahurira, Dedan Okello, Joan Wamulugwa, Lawrence Ssegawa, Julius Wandabwa, Sarah Kiguli, Martin Chebet, Milton W. Musaba

**Affiliations:** 1 Department of Community and Public Health, Busitema University, Mbale, Uganda; 2 Department of Research, Nikao Medical Center, Kampala, Uganda; 3 Department of Obstetrics and Gynecology, Busitema University, Mbale, Uganda; 4 Busitema University Centre of Excellency for Maternal and Child Health, Mbale, Uganda; 5 Makerere University Hospital, Makerere University Kampala, Kampala, Uganda; 6 Mbale Regional Referral Hospital, Mbale, Uganda; 7 Department of Paediatrics and Child Health, Busitema University, Mbale, Uganda; 8 Department of Research, Sanyu Africa Research Institute, Mbale, Uganda; 9 Department of Paediatrics and Child Health, Makerere University, Kampala, Uganda

**Keywords:** Renal function, Children, Obstructed labour, eGFR, Uganda

## Abstract

**Background:**

Over two million children and adolescents suffer from chronic kidney disease globally. Early childhood insults such as birth asphyxia could be risk factors for chronic kidney disease in later life. Our study aimed to assess renal function among children aged two to four years, born to women with obstructed labour.

**Methods:**

We followed up 144 children aged two to four years, born to women with obstructed labor at Mbale regional referral hospital in Eastern Uganda. We used serum creatinine to calculate estimated glomerular filtration rate (eGFR) using the Schwartz formula. We defined decreased renal function as eGFR less than 90 ml/min/1.73m^2^.

**Results:**

The mean age of the children was 2.8 years, standard deviation (SD) of 0.4 years. Majority of the children were male (96/144: 66.7%). The mean umbilical lactate level at birth among the study participants was 8.9 mmol/L with a standard deviation (SD) of 5.0. eGFR of the children ranged from 55 to 163 ml/min/1.73m^2^, mean 85.8 ± SD 15.9. Nearly one third of the children (45/144) had normal eGFR (> 90 ml/Min/1.73m^2^), two thirds (97/144) had a mild decrease of eGFR (60–89 ml/Min/1.73m^2^), and only two children had a moderate decrease of eGFR (< 60 ml/Min/1.73m^2^). Overall incidence of reduced eGFR was 68.8% [(99/144): 95% CI (60.6 to 75.9)].

**Conclusion:**

We observed a high incidence of reduced renal function among children born to women with obstructed labour. We recommend routine follow up of children born to women with obstructed labour and add our voices to those calling for improved intra-partum and peripartum care.

**Supplementary Information:**

The online version contains supplementary material available at 10.1186/s12882-024-03552-8.

## Introduction

Over 850 million people and at least 2 million children and adolescents are affected by chronic kidney disease worldwide [1–3]. Low and middle income countries contribute 70% of the global burden of chronic kidney disease [4]. The prevalence of chronic kidney disease is estimated to range from 10.1 to 15.8% in Africa. West and central Africa have the highest prevalence of chronic kidney disease at 16%, followed by Southern Africa (12.2%) and East Africa (11%) [5]. The prevalence of chronic kidney disease in Uganda (14%) is higher than regional estimates [6].

The age specific mortality rate among children with chronic kidney is 30 to 150 times higher compared to children without kidney disease [7]. Mortality among children with chronic disease ranges from 17 to 20% [7, 8]. More than 40% of children diagnosed with chronic disease in developing countries present late with kidney failure [9]. This can be attributed to low awareness about risk factors for chronic kidney disease among practitioners hence the untimely diagnosis and referral [10]. Early detection and appropriate management of children at risk of developing chronic kidney disease can prevent or slow disease progression to kidney failure [9]. Early identification of children with reduced renal function would alleviate high costs associated with management of chronic kidney disease in later life [10]. There is urgent need to identify risk factors for chronic kidney disease among children in developing countries, so that timely and effective interventions are instituted.

The life course epidemiology approaches suggest that insults in the peripartum period could result in non-communicable diseases such as chronic kidney disease in later life [11]. One potential insult in the peripartum period is obstructed labour. Obstructed labour complicates 22% of pregnancies [12], and is involved in 50% of perinatal deaths [13] and 9% of maternal deaths [12] in Uganda. Unrelieved obstruction is associated with prolonged fetal asphyxia, which results in shunting of blood from the kidneys to maintain cerebral, cardiac, and adrenal perfusion [14]. Under-perfusion of the kidney is followed by necrotic and apoptotic cell injury to the cortical and tubular renal parenchymal cells hence acute kidney injury [15]. Recovery from acute kidney injury by maladaptive repair results in alteration of the kidney structure hence irreversible damage [15–17].

Recent advances in chronic kidney disease highlight the role of neonatal insults in its pathogenesis [11]. Approximately 27–67% of survivors of neonatal acute kidney injury present with reduced renal function at two to three years [15, 18, 19]. However, it is not clear whether the kidney damage following obstructed labour persists into infancy. Early identification of renal dysfunction could result in timely interventions which could be life and organ saving. Therefore, we aimed to assess renal function among children aged 2 to 4 years, born to women with obstructed labour in Mbale regional referral hospital.

## Methods

### Study design

We conducted a cohort study among children aged two to four years, born to women with obstructed labour. These women had participated in a double blind, randomized controlled trial to establish the effect of sodium bicarbonate on maternal and perinatal outcomes among women with obstructed labour in Mbale regional referral hospital between July 2018 and September 2019 [20], **Trial registration number;** PACTR201805003364421.

### Study setting

We conducted the study in Mbale regional referral and teaching hospital between October 2021 to April 2022. Mbale regional referral hospital is a public hospital that serves four district hospitals and 10 health sub-districts in and around Elgon sub-region (with about 4 million people). The hospital is staffed by 4 pediatricians, runs 4 special clinics: diabetic clinic, pediatric psychiatry clinic, neonatal clinic and a chronic care clinic. The chronic care clinic runs every Wednesday for children with sickle cell disease, kidney disease, heart disease. Annually, about 5% (600) of the women that deliver in Mbale regional referral hospital are diagnosed with obstructed labour [21].

### Study participants

We recruited children aged 2 to 4 years, born to women with obstructed labour. These women participated in a clinical trial between July 2018 and September 2019 to assess the effect of a sodium bicarbonate infusion on blood lactate, maternal and perinatal outcomes among women with obstructed labour in Mbale regional referral hospital [20].

### Inclusion criteria

We included children aged 2 to 4 years born to women with obstructed labour in the PACTR201805003364421 trial [20], alive and willing to come to Mbale regional referral hospital for follow up.

### Exclusion criteria

We excluded children who were too sick to participate in the study.

### Outcome variables

Our outcome of interest was renal function defined as estimated glomerular filtration rate (eGFR). We used serum creatinine values to calculate eGFR using the Schwartz formula in Stata: *egfr, f(schwartz) cr(SCreatinine) s height(average height of child)* [22]. We defined normal eGFR as greater than or equal to 90 ml/min/1.73m^2^. We defined reduced eGFR as less than 90 ml/min/1.73m^2^. We defined mild decrease of eGFR as 60 to 89 ml/min/1.73m^2^ while moderate decrease of eGFR was defined as eGFR less than 60 ml/min/1.73m^2^.

#### Independent variables

We considered the following:

1. Perinatal characteristics of the child (birth weight, gestational age, Apgar score, umbilical arterial lactate, neonatal illness).
2. Socio demographic characteristics of the child (sex, age).
3. Nutrition history of the child (early and exclusive breast feeding, food diversity, stunting, wasting, and meal frequency).
4. Medical history of the child (HIV status, admission for malaria or any illness in past one year, blood pressure measurement).
5. Socio demographic characteristics of the mother (maternal age at birth, wealth index).

#### Data collection

Once informed consent was provided and eligibility confirmed, the following information and specimens were obtained:

1. Clinical history included assessment of the nutrition and past medical history of the child. Socio demographic characteristics of the child and mother were also obtained during history taking.
2. Physical examination included a general body examination, and anthropometric measurements (height, weight, mid upper arm circumference and head circumference) according to WHO/UNICEF guidelines.
3. The caretaker was given a urine bottle and instructed on how to collect a midstream sample.
4. Under aseptic conditions, we obtained approximately 3mls of blood using a single-use 5 ml syringe by venipuncture. The cubital fossa was cleaned with alcohol swabs prior to blood collection. Blood for Serum creatinine was collected in a red top vacutainer and analyzed using a COBAS-INTEGRA 400 machine and at the lancet laboratories in Mbale (South African National Accreditation System Reg. No: 1996/006959/07). HIV tests were carried out according to Ministry of Health testing procedures using two rapid enzyme linked immunoassays (Determine HIV-1/2, Abbott Laboratories, USA; Uni-Gold, Trinity Biotech PLC, Ireland) and a third rapid test (HIV 1/2 STAT-PAK, Chembio diagnostics, USA) if results were discordant. A drop of blood was put on the Random Blood Sugar Kit. Random blood glucose was measured in mmol/L using On Call® Plus glucometer (ACON Laboratories, Inc., 10,125 Mesa Road, San Diego, California, USA).

### Sample size and sampling

The sample size was dependent on the size of the parent follow up study whose aim was to determine the prevalence of neurodevelopmental delay [23]. A total of 144 participants had a serum creatinine measurement. This sample size resulted in an absolute precision of 2.3–8.2%, i.e. the difference between the point estimate and the 95% confidence interval (CI) for prevalence values ranging from 2 to 50%, a precision we deemed adequate.

### Statistical analysis

Data were analyzed using Stata version 14.0 (Stata Corp; College Station, TX, USA). Continuous variables were summarized into means, median, and standard deviation. Categorical variables were summarized into proportions. Reduced eGFR was defined as eGFR less 90 ml/min/1.73m^2^. Bivariable and multivariable analyses were done using generalized linear model for the Poisson family with a log link to obtain prevalence ratios to assess the strength of associations between selected exposure variables and reduced eGFR. The selected exposure variables were based on literature review and included maternal age [24], birthweight [25], mode of delivery [26], age of child [27], sex [27], blood pressure [28], nutrition [29] and severe malaria [30].

### Bias analysis

A total of 393/537 of participants recruited at baseline did not return for follow up. To estimate the effect of this missingness on our study results, we conducted multiple imputations using Stata v14.0 The missing variable was reduced eGFR, binary in nature, so we used the logistic regression for a binary variable imputation method. We assumed that our data was missing completely at random. For the imputation, we included variables with less than 10% missingness collected at baseline: Apgar at one-minute, maternal age at time of birth, birth weight of the baby, mode of delivery, arterial lactate level, religion, marital status, maternal education level, maternal occupation, residence, history of taking alcohol, HIV status, level of education of the partner, occupation of the partner, parity, number of antenatal care visits and referral status. We also conducted logistic regression analyses to identify factors associated with failure to come for follow up. We imputed 20 datasets. We obtained one set of statistical results on each imputed data set and combined them using Rubin’s rule. We used Stata’s *mi estimate: proportion var* command to calculate the combined incidence. We also compared baseline characteristics of children who came for follow up and those who did not come for follow up. This has been presented in appendix 2.

## Results

### Study profile

**Fig. 1:**
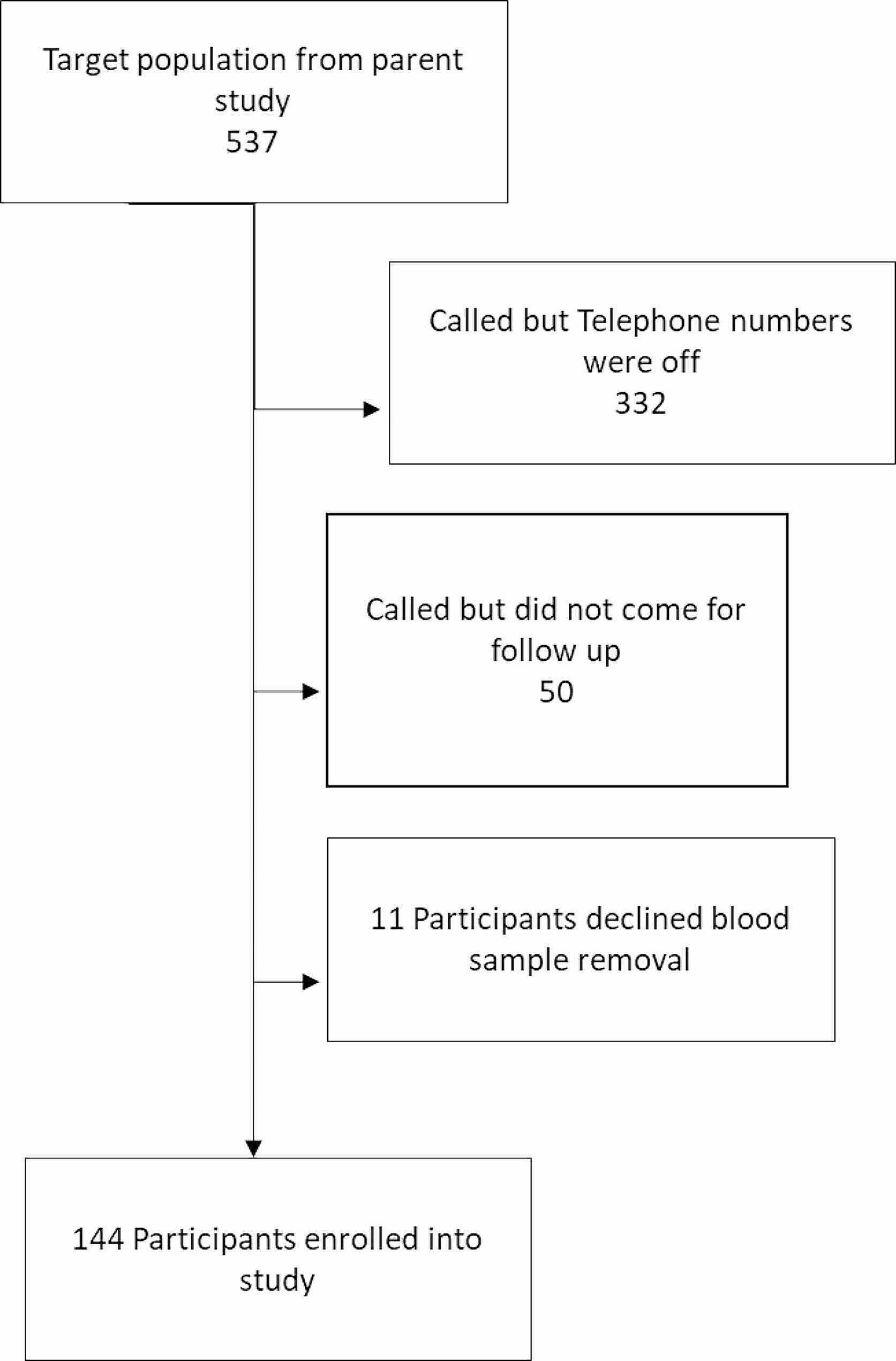
Participants in a follow up study of children born to women with obstructed labour in Eastern Uganda

In the parent study, we enrolled 537 women diagnosed with obstructed labour. Out of these, 155 brought their children for follow up and 144 consented to have blood samples picked from their children for analysis. Details of patient flow are indicated in Fig. 1.

### Participant characteristics

We enrolled 144 children with a mean age of 34.0 months standard deviation (SD) of 4.6 months. Most of the participants were male 96/144 (66.7%), majority had normal birth weight 138/144 (96%), all were born at term, most the participants 141/144 (98%) had an Apgar score of 7 and above at one minute, only 58/144 (40.3%) were exclusively breastfed. The mean umbilical lactate level at birth among the study participants was 8.9 mmol/L with a standard deviation (SD) of 5.0. Tables 1 and 2 summarize the socio-demographic, characteristics, nutrition history, perinatal characteristics, and past medical history of children born to women with obstructed labour in Eastern Uganda.

**Table 1:** Socio demographic characteristics and nutrition history of children born to women with obstructed labour in Eastern Uganda

	Normal eGFR *N* = 45 n (%)	Reduced eGFR *N* = 99 n (%)	Total *N* = 144 n (%)	P values
Sex				
Male	26(57.8)	70(70.7)	96(66.7)	0.13
Female	19(42.2)	29(29.3)	48(33.3)	
**Current age of the child**				
2 to less than 3 years	26(57.8)	66(66.7)	92(63.9)	0.3
3 to 4 years	19(42.2)	33(33.3)	52(36.1)	
**Child’s birth order**				
First born	19(42.2)	50(50.5)	69(47.9)	0.28
02-Apr	16(35.6)	37(37.4)	53(36.8)	
05-Dec	10(22.2)	12(12.1)	22(15.3)	
**Early breastfeeding** **initiation**				
No	24(53.3)	63(63.6)	87(60.4)	0.24
Yes	21(46.7)	36(36.4)	57(39.6)	
**Exclusive breastfeeding**				
No	23(51.1)	63(63.6)	86(59.7)	0.16
Yes	22(48.9)	36(36.4)	58(40.3)	
**Food diversity**				
No	3(6.7)	15(15.2)	18(12.5)	0.15
Yes	42(93.3)	84(84.8)	126(87.5)	
**Stunting**				
No	37(82.2)	78(78.8)	115(79.9)	0.63
Yes	8(17.8)	21(21.2)	29(20.1)	
**Wasting**				
No	44(97.8)	93(93.9)	137(95.1)	0.32
Yes	1(2.2)	6(6.1)	7(4.9)	
**Minimum meal frequency**				
< 4 meals a day	12(26.7)	33(33.3)	45(31.3)	0.42
4 and above meals	33(73.3)	66(66.7)	99(68.7)	
**Wealth index**				
Q1. Poorest	9 [20]	20(20.2)	29(20.2)	0.95
Q2	10(22.2)	20(20.2)	30(20.8)	
Q3	9 [20]	21(21.2)	30(20.8)	
Q4	10(22.2)	18(18.2)	28(19.4)	
Q5. Richest	7(15.6)	20(20.2)	27(18.8)	

**Table 2:** Perinatal and clinical characteristics of children born to women with obstructed labour in Eastern Uganda

	Normal eGFR *N* = 45 n (%)	Reduced eGFR *N* = 99 n (%)	Total *N* = 144 n (%)	P values
*Birth weight				
Normal birth weight > = 2.5 kg	44(98)	94(97)	138(96)	0.77
Low birth weight < 2.5 kg	1 [2]	3 [3]	4 [4]	
**Gestational age**				
37 weeks	9 [20]	10 [10]	19 [13]	0.1
More than 37 weeks	36(80)	89(90)	125(87)	
**Apgar at one minute**				
Less than 7	0(0)	3 [3]	3 [2]	0.24
7 and above	45(100)	96(97)	141(98)	
**Apgar at five minutes**				
Less than 7	613)	14 [14]	20 [14]	0.9
7 and above	39(87)	85(86)	124(86)	
*Umbilical arterial lactate (mmol/L)				
< 4.8	10(23.3)	21(21.2)	31(21.8)	0.96
4.8–10	19(44.2)	46(46.5)	65(45.8)	
> 10	14(32.6)	32(32.3)	46(32.4)	
**Admission to neonatal Unit**				
No	36(80)	85(85.9)	121(84)	0.37
Yes	9 [20]	14(14.1)	23 [16]	
**Oxygen after birth**				
No	39(86.7)	91(91.9)	130(90.3)	0.32
Yes	6(13.3)	8(8.1)	14(9.7)	
**Admission for any illness** **in the last one year**				
No	34(75.6)	86(86.9)	120(83.3)	0.91
Yes	11(24.4)	13(13.1)	24(16.7)	
**Admission for severe** **malaria in last one year**				
No	35(77.8)	88(88.9)	123(85.4)	0.8
Yes	10(22.2)	11(11.1)	21(14.6)	
**HIV status**				
Negative	38(84.4)	91(91.9)	129(89.6)	0.17
Positive	7(15.6)	8(8.1)	15(10.4)	
*Blood pressure				
Normal blood pressure	9 [20]	23 [24]	32((22.9)	0.23
High normal blood pressure	0 (0)	5 [5]	5(3.6)	
Elevated blood pressure	36(80)	67 (71)	103(73.5)	
**Age of mother at time of giving birth**				
16 to 19 years	6 [13]	18 [18]	24 [17]	0.61
20 to 30 years	32(71)	62(63)	94(65)	
More than 30 years	7 [16]	19 [19]	26 [18]	

*Denotes N less than 144

### Reduced eGFR among children born to women with obstructed labour

Overall incidence of reduced eGFR was 68.8% [(99/144) 95% CI (60.6 to 75.9)]. After multiple imputation, the incidence of reduced eGFR among children able to come for follow up was 66.1%, [95% CI (55.9-76.4%)]. Of the 144 children that were followed up 45 (31.2%) had normal eGFR (> 90 ml/min/1.73m^2^), 97 (67.4%) had mild decrease of eGFR (60–89 ml/min/1.73m^2^). Only 2 (1.4%) had a moderate decrease of eGFR (< 60 ml/min/1.73m^2^). eGFR ranged from 55 ml/min/1.73m^2^ to 163 ml/min/1.73m^2^, mean eGFR was 85.77mmol/L ± SD 15.9 and median eGFR was 83.8mmol/L.

Children with elevated blood pressure were 26% more likely to have reduced renal function compared to children with normal blood pressure adjusted prevalence ratio (APR) 1.26 [95% CI (1.00-1.60)]. Children with higher umbilical arterial lactate had a higher risk of reduced renal function compared to children with lower higher umbilical arterial lactate. APR 1.14 [95% CI (0.87–1.49)]. Table 3 summarizes factors associated with reduced eGFR.

**Table 3:** Factors associated with reduced eGFR among children born to women with obstructed labour in Eastern Uganda

	Crude Prevalence Ratio and 95% CI	Adjusted Prevalence Ratio and 95% CI	P value
Maternal age			
	1	1	
17 to 19 years	1.11(0.85–1.45)	1.22(0.94–1.58)	0.142
**Birth weight**			
Normal birth weight	1	1	
low birth weight	1.1(0.62–1.97)	1.18(0.62–2.27)	0.611
**Mode of delivery**			
Vaginal delivery	1	1	
Cesarean section	0.9(0.52–1.57)	0.92(0.58–1.47)	0.732
**Age of child at follow up**			
3 to 4 years	1	1	
2 to < 3 years	1.13(0.89–1.44)	1.18(0.91–1.53)	0.21
**Sex of child**			
Female	1	1	
Male	1.21(0.93–1.57)	1.21(0.90–1.63)	0.198
**Umbilical artery lactate**			
0 to 4.8mmol	1	1	
> 4.8mmol	1.04(0.79–1.36)	1.14(0.87–1.49)	0.354
**Blood pressure of child** **at follow up**			
Normal blood pressure	1	1	
Elevated blood pressure	1.19(0.95–1.49)	1.26(1.00-1.60)	0.053
**Stunting of child**			
No	1	1	
Yes	1.07(0.82–1.38)	0.96(0.72–1.29)	0.79
**Wealth index of caregiver**			
1st quintile	1	1	
2nd quintile	0.97(0.68–1.38)	1(0.69–1.46)	0.984
3rd quintile	1.01(0.72–1.43)	0.94(0.63–1.39)	0.739
4th quintile	0.93(0.64–1.35)	1.03(0.71–1.49)	0.882
5th quintile	1.07(0.77–1.50)	1.15(0.79–1.65)	0.467
**Exclusively breast-fed child**			
Yes	1	1	
No	1.18(0.93–1.50)	1.13(0.85–1.49)	0.401
**Hospitalised for malaria** **in last year**			
No	1	1	
Yes	0.73(0.48–1.12)	0.66(0.42–1.05)	0.078
**Food diversity of child’s diet**			
Yes	1	1	
No	1.25(0.98–1.59)	1.12(0.84–1.49)	0.446

## Discussion

Our study found a high incidence of decreased renal function in this cohort [68.8% (99/144)] of children born to women with obstructed labour. This can be explained by the fact that hypoxia experienced by a newborn could result in renal injury as previously described [15–17]. Our findings are consistent with a study done in British Columbia among 126 children less than one year admitted with acute kidney injury. The surviving children were assessed for risk of chronic kidney disease at 1 year, 2 years or 3 years and 60% had mildly decreased eGFR of 60–90 mL/min/1.73m^2^ [19]. However, our results need to be interpreted with caution due to the high loss to follow up. We conducted a bias analysis using multiple imputation and found similar results. Our incidence was much higher as compared to a retrospective study in the USA which followed up 80 children with nephrotoxic acute kidney injury secondary to aminoglycosides which found that only 23% had an eGFR < 90 mL/min/1.73 m^2^. This can be explained by the fact that this study involved much older children without history of birth asphyxia [31].

Children who had elevated blood pressure were 26% more likely to have reduced renal function compared to children with normal blood pressure. The elevated blood pressure could have been caused by alterations in fluid and electrolyte balance following reduced renal function in this population [25, 32]. Possible reasons for elevated blood pressure in this cohort have been reported and discussed by Mukunya et al. [28].

Our study found a tendency of children with higher umbilical lactate having greater risk of renal dysfunction. This finding was imprecise due to the small sample size. However, almost all children in our cohort had abnormally high levels of lactate as evidenced by the high mean arterial lactate level of 8.9 mmol/L which is above the normal (≤ 4.8 mmol/L). This can explain the absence of a dose response because almost all children in our cohort might have been exposed to the “minimum dose” of birth asphyxia needed to elicit renal dysfunction. Other studies that used Apgar score and grade of hypoxic-ischemic encephalopathy as markers of degree of asphyxia found a correlation between severity of asphyxia and renal impairment at 96 h of life [33] and 6 months of age [34]. We believe umbilicate arterial lactate is a more accurate measure of birth asphyxia.

Our study did not find an association between age of the child, low birth weight, admission for malaria, male sex and reduced eGFR. This could be due to our small sample size. However other studies have found all the above to be associated with reduced renal function in infancy [25, 27, 30].

### Strength and limitations

This was a follow up study to assess the renal function in a special population of children born to women with obstructed labour. A major limitation of our study is the high loss to follow up. When we compared baseline characteristics of children who came for follow up to those of children who did not come for follow up, children who did not come for follow up had: − 1) a higher median arterial lactate, 2) a higher proportion with an Apgar less than 7, and 3) a higher proportion born to teenage mothers. These observations point to higher morbidity and possible subsequent mortality among children not followed up. As such, we could have under-estimated the burden of renal dysfunction in this group.

There is uncertainty regarding the accuracy of the Schwartz formula which has been shown to overestimate true eGFR by as much as 25-30% [35]. Furthermore, our study did not collect data on history of use of nephrotoxic medication and baseline urine creatinine at birth was not determined. Therefore, we were unable to assess for history of neonatal AKI, and other possible causes of neonatal AKI in this cohort. In addition, we neither monitored renal function to assess for chronicity nor collected urine creatinine levels at follow up, as such we were unable to determine albumin to creatinine ratio which is a good marker of kidney function. Other limitations include the fact that renal ultrasound to rule out congenital abnormalities of the kidney and urinary tract (CAKUT) was not done, as well as our lack of a comparison group of babies born to women who did not have obstructed labour.

## Conclusion

We observed a high incidence of reduced renal function among children born to women with obstructed labour. We recommend routine follow up and screening for renal dysfunction among children born to women with obstructed labour. We recommend further studies designed to follow up trends of renal function in children who are born with asphyxia. Finally, we add our voices to those calling for improved intra-partum and peripartum care.

### Electronic supplementary material

Below is the link to the electronic supplementary material.

Supplementary Material 1

## Data Availability

The datasets used and/or analyzed during the current study are available from the corresponding author.

## Abbreviations

CKD: Chronic kidney disease
AKI: Acute kidney injury
MRRH: Mbale regional referral hospital
EGFR: Estimated glomerular filtration rate
APR: Adjusted Prevalence Ratio
