# Supplementary material for: Decreased renal function among children born to women with obstructed labour in Eastern Uganda: a cohort study
Source: BMC Nephrol. 2024 Mar 28;25:116. doi: 10.1186/s12882-024-03552-8 (PMC10976667; doi:10.1186/s12882-024-03552-8)
Supplement: Supplementary file 1 — Supplementary Material 1 [file 12882_2024_3552_MOESM1_ESM.docx]

Appendices.

**Appendix 1**

**Table 4: Urine dipstick results of children born to mothers with obstructed labour**

|  | Normal eGFR N=45  n | Reduced eGFR N=99  n |
| --- | --- | --- |
| **Urine glucose** |  |  |
| Positive | 0 | 0 |
| Negative | 44 | 95 |
| *Missing | 1 | 4 |
| **Urine protein** |  |  |
| Positive | 1 | 3 |
| Negative | 43 | 94 |
| *Missing | 1 | 2 |
| **Red blood cells in urine** |  |  |
| Positive | 3 | 7 |
| Negative | 41 | 90 |
| *Missing | 1 | 2 |

*Missing means not done

**Appendix 2**

**Table 5. Comparing perinatal characteristics of children who came for follow up with children who did not come for follow up.**

|  | Did not come for follow up N=393 n (%) | Came for follow up  N=144 n (%) | P value |
| --- | --- | --- | --- |
| **Apgar score at one minute** |  |  |  |
| Median (IQR) | 8 (6 to 9) | 8 IQR (7 to 9) |  |
| ***Apgar at 1 minute** |  |  |  |
| Apgar <7 | 120 (30.6%) | 20 (13.9%) | <0.001 |
| Apgar >=7 | 272 (69.4%) | 124 (86.1%) |  |
| **Apgar score at 5 minutes** |  |  |  |
| Median (IQR) | 10 (8 to 10) | 10 IQR (10 to 10) |  |
| ***APGAR at 5 minutes** |  |  |  |
| Apgar <7 | 57 (14.5%) | 3 (2.1%) | <0.001 |
| Apgar >=7 | 335 (85.5%) | 141 (97.9%) |  |
| **Arterial lactate** |  |  |  |
| Median (IQR) | 9.2 IQR (5.4 to 14.3) | 7.2 IQR (5.3 to 11.4) |  |
| ***Arterial lactate** |  |  |  |
| min-4.8mmol/L | 75 (20.0%) | 31 (21.8%) | 0.65 |
| >4.8 mmol/L | 300 (80.0%) | 111 (78.2%) |  |
| ***Age of mother** |  |  |  |
| 17-19 years | 129 (32.9%) | 24 (16.7%) | <0.001 |
| 20-30 years | 217 (55.4%) | 94 (65.3%) |  |
| >30 years | 46 (11.7%) | 26 (18.1%) |  |
| ***Birth weight** |  |  |  |
| Low birth weight | 11 (2.8%) | 4 (2.8%) | 1.00 |
| Normal birth weight | 379 (97.2%) | 138 (97.2%) |  |

*Denotes N less than 537
